# Review of Denonvilliers’ fascia: the controversies and consensuses

**DOI:** 10.1093/gastro/goaa053

**Published:** 2020-09-30

**Authors:** Xiao-Ming Zhu, Guan-Yu Yu, Nan-Xin Zheng, Hui-Min Liu, Hai-Feng Gong, Zheng Lou, Wei Zhang

**Affiliations:** Department of Colorectal Surgery, Changhai Hospital, Shanghai, P. R. China; Department of Colorectal Surgery, Changhai Hospital, Shanghai, P. R. China; Department of Colorectal Surgery, Changhai Hospital, Shanghai, P. R. China; Colorectal Surgery Service, Department of General Surgery, Tan Tock Seng Hospital, Singapore; Department of Colorectal Surgery, Changhai Hospital, Shanghai, P. R. China; Department of Colorectal Surgery, Changhai Hospital, Shanghai, P. R. China; Department of Colorectal Surgery, Changhai Hospital, Shanghai, P. R. China

**Keywords:** Denonvilliers’, fascia, rectovaginal septum, rectal cancer, total mesorectal excision

## Abstract

The Denonvilliers’ fascia (DVF) plays an important role in rectal surgery because of its anatomic position and its relationship to the surrounding organs. It affects the surgical plane anterior to the rectum in the procedure of total mesorectal excision (TME). Anatomical and embryological studies have helped us to understand this structure to some extent, but many controversies remain. In terms of its embryonical origin, there are three mainstream hypotheses: peritoneal fusion of the embryonic cul-de-sac, condensation of embryonic mesenchyme, and mechanical pressure. Regarding its architecture, the DVF may be a single, two, or multiple layers, or a composite single-layer structure. In women, most authors deem that this structure does exist but they are willing to call it the rectovaginal septum rather than the DVF. Operating behind the DVF is supported by most surgeons. This article will review those mainstream studies and opinions on the DVF and combine them with what we have observed during surgery to discuss those controversies and consensuses mentioned above. We hope this review may help young colorectal surgeons to have a better understanding of the DVF and provide a platform from which to guide future scientific research.

## Introduction

It has been 180 years since Denonvilliers [1] first discovered a distinct and clear dartoid membranous layer between the seminal vesicle, prostate, and rectum. He called it the ‘prostato-peritoneal membrane’ during anatomical dissection on 12 male cadavers. This observation was submitted as his doctoral thesis to the University of Paris in the following year [2]. Thereafter, this structure was called the Denonvilliers’ fascia (DVF) by next generations in memory of his findings. The DVF is in the narrow pelvic cavity and is adjacent to the rectum, vagina, prostate, seminal vesicle, and pelvic plexus nerves, which makes it difficult to study. In the past, many anatomical, histological, and embryological studies have shown us details about the DVF, although many controversies about its origin, anatomy, and the relation with surgery remain. This article will review these controversies and consensuses, and we hope that those previous pieces of research may help us to re-recognize this structure and provide a platform from which to guide future scientific research of the DVF.

## The embryological origin of the DVF

The embryological origin of the DVF has been debated since it was discovered. To date, three main hypotheses explain its embryological origin: peritoneal fusion of the embryonic cul-de-sac, condensation of embryonic mesenchyme, and mechanical pressure.

The fusion theory was suggested by Cunéo and Veau [3] in 1899. They proposed that the embryonic peritoneum in the rectovesical or rectouterine cul-de-sac came close to each other and then fused to form the DVF. This theory was supported by Smith [4, 5] based on his anatomical dissection of adults and fetuses. However, Wesson [6] doubted the fusion theory based on his research. He discussed that, with the development of the fetus, the two sides of the embryonic cul-de-sac gradually came close to each other and faded away, and were replaced by mesenchymal tissue, which gradually condensed to form the DVF. Therefore, he proposed the mesenchymal-condensation theory. Contemporaneously, Tobin and Benjamin [7] supported the fusion theory by their persuasive evidence on the mesenchymal tissue described by Wesson, which could indeed be seen between the rectum and the genitourinary organs at different embryonic stages, but these tissues differentiated into muscles and connective tissue eventually. They found that these mesenchymal tissues were surrounded by a layer of mesothelial cells, which were the continuation of the peritoneum from the cul-de-sac. These mesenchymal tissues disappeared after fusion, leaving behind a layer of fibrous-tissue membrane to form the DVF. However, Silver [8] denied the fusion theory based on his findings. His study showed that the cul-de-sac disappeared at the 8th week of gestation and was replaced by smooth muscles. Kim *et al*. [9] believed that these smooth muscles that ‘untimely appeared’ during the embryonic period were recognized as membranous structures mistakenly. In their research, there was no linear arrangement of mesenchymal cells to form a clear fascia structure after the disappearance of the cul-de-sac and this status lasted until the 16th week of gestation. This status was extended to 20 weeks by Kraima *et al*. [10] and the explicit fascia was recognized at the 24th week of gestation by Fritsch and Kühnel [11]. Therefore, Kim *et al*. [9] proposed the pressure theory that the mesenchymal tissue was extruded to form the DVF under the mechanical pressure that was produced by the gradually enlarged rectum, seminal vesicle, prostate, or vagina.

As we can see, different authors have different opinions supported by their own evidence. In view of the fact that there is no evidence of peritoneal fusion in the embryological studies, most authors support the condensation-of-embryonic-mesenchyme theory at present. But there is still no agreement on how the DVF is actually formed and this still needs further research.

## The architecture of the DVF

Here is Denonvilliers’ original description of the DVF translated by Chapuis *et al*. [12]:

‘Behind the prostate, between the seminal vesicles and the rectum, there is a clear and distinct membranous layer, which I call the prostato-peritoneal membrane. Here then is its arrangement. On either side, it merges with the dense cellular tissue which surrounds and sheathes the inferior vesical venous plexus at the base of the bladder; anteriorly, it blends with the most backward extremity of the prostate; posteriorly, it is adherent to that portion of the peritoneum which descends between the bladder and the rectum. The texture of this membranous layer is akin to that of the dartos; it is made up principally of fibers which fan out radially such that those arising from the posterior midline are the most pronounced; in well-developed subjects muscle fibers exist but only on either side of the fascia.’

The DVF seems to be a single layer that encloses the prostate and seminal vesicle according to his description. However, other authors had different ideas. Smith [5] and Wesson [6] proposed that the DVF consisted of two layers, whereas Tobin and Benjamin [7] described the propria fascia of the rectum as the posterior layer of the DVF, although these two fasciae have different embryonic origins. These various descriptions made many surgeons confused about the real concept of the DVF and its relationship with rectal surgery, while it also urges future investigators to carry out further research.

Kourambas *et al*. [13] presented their findings on cadavers in 1998, demonstrating that the DVF was a single layer with no distinct lateral border and was adhered closer to the prostate anteriorly. It formed an ‘H’-shaped structure with the surrounding pelvic fascia and perirectal fascia between the prostate and rectum. In the same year, Nano *et al*. [14] described a clear space between the two layers of DVF through cadaveric and embryonic studies, in which the anterior layer was closer to the prostate and the posterior layer was separated from the rectum by loose connective tissue. Later, Lindsey *et al*. [15] concluded that the DVF was a single-layer membrane formed by the fusion of the peritoneum during the embryonic period after he reviewed 115 relevant articles. Ludwikowski *et al*. [16] studied 63 fetal specimens from the 9th week of gestation to birth. His results revealed that the original DVF gradually formed from the caudal perineal body to the peritoneal fold from the 9th week of gestation and finally developed into a single-layer membrane. Bilaterally, it extended to the connective tissue containing the blood vessels and nerves of the genitourinary organs. In his study, the DVF consisted mainly of collagen fibers, although some smooth-muscle fibers could be also seen in the caudal part, which originated from the longitudinal muscle layer of the rectum, and these smooth-muscle fibers could not be considered a component of the DVF. His compatriot Aigner *et al*. [17] confirmed these findings through a similar study later, but he concluded that the DVF not only consisted of collagen fibers, but also contained elastic fibers and smooth-muscle tissue. These findings were consistent with the results of later research [18].

However, Kinugasa *et al*. [19] found that the DVF was a monolayer composite structure in his histological study on 10 male cadavers. His results prompted that the DVF was a monolayer in the center and divided into two or three layers laterally on both sides, some of which ended in neurovascular bundles and some extended to the posterolateral side, separating the neurovascular bundle from the mesorectum clearly. Bertrand *et al*. [20] used 3D reconstruction technology to simulate details of the DVF based on the anatomical dissection of a female fetus: multilayer fascia was tight in the center and loose in the bilateral side, which formed a ‘Y’ -shaped structure, whereas Zhang *et al*. [21] held different views. He considered that the DVF was divided into the anterior layer, which continued with the presacral fascia, and the posterior layer, which continued with the propria fascia of the rectum. The two-layer annular structure surrounded the rectum and formed the perirectal space.

It can be seen that the controversies surrounding the architecture of the DVF are similar concerning its embryonic origin and, although there are many related studies, none of them can provide concrete evidence to help us to reach a consensus on the real structure and route of the DVF. Maybe just like Kim *et al*. [9] speculated in their pressure theory, the DVF is so multifarious that it can appear as different forms in different periods, ages, or genders.

## The DVF in females

The DVF was first found in male cadavers and was widely accepted by surgeons. Compared to men, Denonvilliers did not describe this structure in detail in women. Since surgeons can rarely find a distinct structure between the rectum and the vagina during surgery that is similar to the DVF in males, the existence of the DVF in females have been doubted. Ricci *et al*. [22] concluded that there were only loose and scattered reticular fibers between the rectum and the vagina from the anorectal junction to peritoneal reflection with no fascia-like structure based on the microscopic observations of 22 female cadavers (4 fetuses, 3 infants, and 15 adults). Meanwhile, in recent years, Kleeman *et al*. [23] and Zhang *et al*. [24] also confirmed that no evidence of membranous structure exists in the space between the rectum and the vagina, which was only filled with adipose tissue, fragmented fibrous tissue, and some muscle fibers.

On the contrary, results of most studies support the existence of the DVF in females. Cunéo and Veau [3] and Silver [8] found that there was a similar structure in women with the same components as in men when they studied the origin of the DVF. This structure in women is more commonly referred to as the rectovaginal septum rather than the DVF. Milley and Nichols [25] considered that the rectovaginal septum was an inherent normal structure in women, which could not be affected by age, hormones, and other factors, after he studied fetal specimens, fresh cadaveric specimens, paraffin-embedded cadaveric specimens, and surgical specimens. Meanwhile, more and more embryological, anatomical, and histological evidence has confirmed the existence of the rectovaginal septum [9, 10, 16, 17, 26]. Accordingly, researchers pay more attention to the origin, component, route, and function of the rectovaginal septum.

With regard to its origin, most researchers believed that the rectovaginal septum was homologous with the DVF in men, which was also included in the three hypotheses mentioned above [10, 24]. As for its component and route, in the early years, Milley and Nichols [25] considered that the rectovaginal septum was a translucent membrane composed of dense connective tissue, which adhered to the posterior wall of the vagina closely. It ended at the peritoneal fold cranially and the perineal body caudally. Bilaterally, it fused with the pelvic parietal fascia. They also postulated that surgeons might ignore or deny its existence because of its inseparable relationship with the vagina. The classic description of the rectovaginal septum is that it is divided into two layers in which the anterior layer is equivalent to the DVF in males and the posterior layer fuses with the propria fascia of the rectum. This description was supported by many authors, but they also debated on its details [26–28].

Zhai *et al*. [29] agreed that the anterior layer was equivalent to the DVF, but he considered that the posterior layer was the propria fascia of the rectum itself. In their description, the rectovaginal septum was composed of the DVF and propria fascia of the rectum. The DVF and propria fascia of the rectum were concomitant in most areas but extended in different directions along the peritoneum cranially instead of ending at the peritoneal fold. The DVF eventually ended at the uterus and the propria fascia of the rectum gradually thinned on the rectal surface until it faded away. Caudally, they gradually separated above the perineal body. The propria fascia of the rectum followed the longitudinal muscle of the rectum to the anorectal junction, while the DVF had different characteristics at different levels: at the cervix and upper vagina, the DVF merged into paravaginal tissue; at the mid vagina, it anchored into the pelvic fascia tendon archi; at the lower vagina, it ended at the lateral outlet of the levator ani muscle.

**Figure 1.**
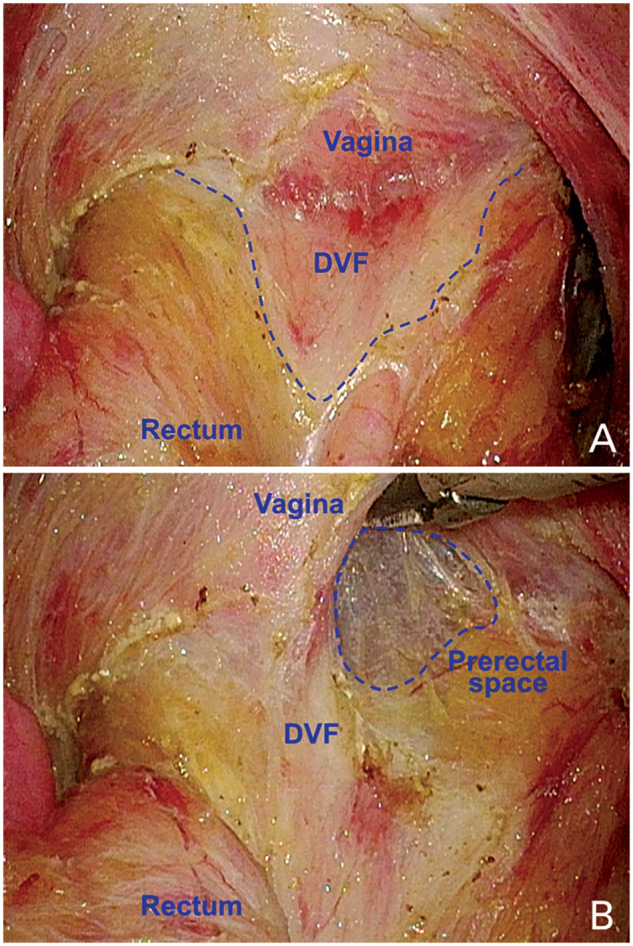
Denonvilliers’ fascia (DVF) in a female during surgery. (A) The DVF is an inverted triangle shape (the blue dotted line). (B) The prerectal space can be seen after cutting the DVF off transversely (the blue dotted circular line).

Meanwhile, with the application of histological methods and 3D-reconstruction technology, the more complex multilayer structure was described. The results of Peschaud *et al*. [30] prompted that the rectovaginal septum was a multilayer structure composed of connective tissue mixed with smooth-muscle fibers from the uterus and vaginal wall. It gradually disappeared into the perineal body caudally and did not connect with the peritoneal fold cranially. It extended anteriorly and posteriorly on the lateral sides to form a ‘Y’-shaped structure. The branches of the ‘Y’ were like an arm covering the neurovascular bundle. In our experience, we have found that nearly 20% of females have a clear and distinct DVF, which presents as an inverted triangle shape behind the vagina during surgery (Figure 1A). After we incise it transversely, we can enter the prerectal space (Figure 1B).

In conclusion, these studies demonstrated that the DVF does exist in women, although there are still many controversies requiring further research. Furthermore, comparing to the controversies on its architecture, investigators have reached a consensus on its function that the rectovaginal septum plays an important role in supporting the pelvic organs, preventing rectocele and vaginal prolapse in females and limiting the spread of inflammation or malignancies [31].

## The relationship between the DVF and rectal surgery

The DVF is so important to colorectal surgeons because it affects the anterior surgical plane of TME in rectal surgery. Heald and Moran [32] concluded that the dissection should be done in front of the DVF for two reasons based on their intraoperative observation: (i) the DVF was closer to the rectum rather than the prostate and it constituted the anterior surface of the mesorectum, which made it difficult to separate; (ii) there was lower local-tumor-recurrence rate compared with dissection behind the DVF [33, 34]. Kraima *et al*. [10] also thought that the surgical plane should be in front of the DVF for achieving the best oncology outcome, although it may injure the urogenital organs or the pelvic plexus.

**Figure 2.**
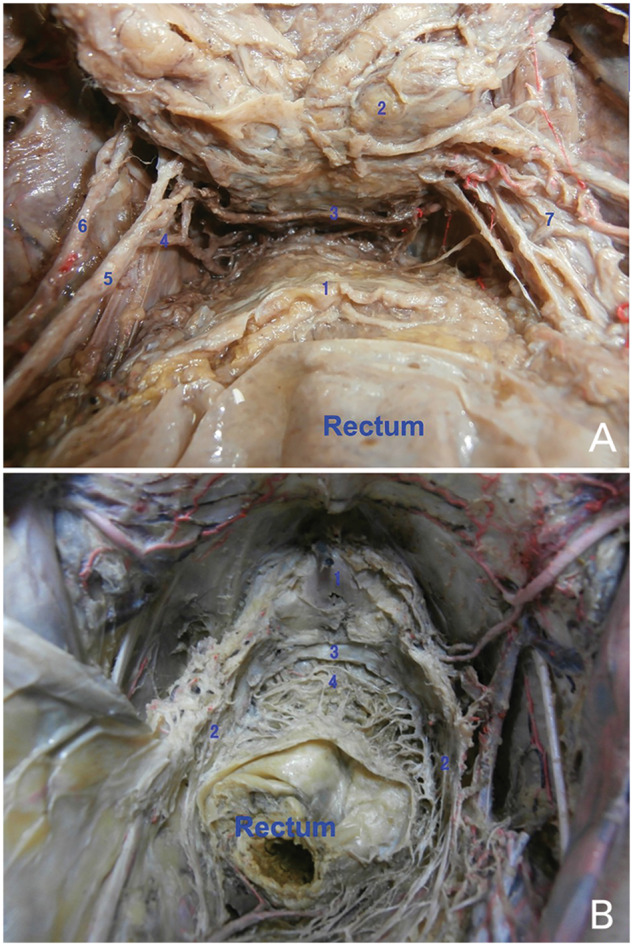
The structures around Denonvilliers’ fascia (DVF) during autopsy. (A) The prostatic branch from the inferior bladder artery (the DVF has been removed). 1, the propria fascia of the rectum; 2, the seminal vesicle; 3, the prostatic branch from the inferior bladder artery; 4, the middle rectal artery; 5, the branch of the anterior trunk of the internal iliac artery; 6, the branch of the obturator artery; 7, the nerve branches from the pelvic plexus. (B) The communicating branches of nerves from the bilateral pelvic plexus (the DVF and prostate have been removed partially). 1, the prostate; 2, the pelvic plexus; 3, the DVF; 4, the communicating branches of the pelvic plexus.

On the contrary, most investigators opined that operating behind the DVF was better based on their studies about its relationship with the pelvic nerves. Taguchi *et al*. [35] and Sugihara *et al*. [36] found that there were many communicating branches coming from the bilateral pelvic plexus on the ventral side of the DVF. These communicating branches were related to urogenital function and, if the nerves were damaged on one side, their function could be compensated for partially by the other side via these branches [35]. Subsequently, Kinugasa *et al*. [19] proved in their histological research that the communicating branches and ganglion cells were connected tightly with the DVF, and some nerve fibers were even embedded in the DVF. Of note, the lateral continuation of the DVF is divided into two or three layers that cover the neurovascular bundle and separate it from the mesorectum. We also noticed this relationship in our previous study. In our anatomic dissection of male cadavers, we could see that many nerve branches from the bilateral pelvic plexus communicating with each other in front of the DVF and the prostatic branch of the inferior bladder artery also could be seen in individual specimens after we removed a part of the prostate (Figure 2) [37]. Kourambas *et al.* [13] had confirmed that damage to these branches was responsible for erectile dysfunction after prostatectomy. Meanwhile, this anatomical relationship was also verified in females [16, 17]. In another study, these nerves and neurovascular bundle covered by the rectovaginal septum were found located at 2 o’clock and 10 o’clock around the mesorectum in females [30].

Lately, by comparing the incidence of post-operative erectile dysfunction between operating behind and in front of the DVF, Liu *et al*. [38] found that the incidence of erectile dysfunction was significantly lower in the group where dissection was done behind the DVF compared to the group in front of the DVF, verifying that injury of the communicating branches in front of the DVF may affect post-operative sexual function. This outcome is consistent with most previous studies that were reviewed by Chapuis *et al*. [12]. They reported that, in 13 out of 16 studies, there was a direct correlation of post-operative sexual function with the DVF, supporting that the optimal surgical plane should be behind the DVF. In our opinion, choosing an optimal surgical plane in front of the rectum is not absolute. It should be determined by both oncology safety and neuroprotection. And oncologic safety should always be the primary consideration compared to other factors. For tumors located on the posterior wall of the rectum, surgeons can operate behind the DVF in order to protect the nerves. However, if the tumor is on the anterior wall and it threatens the safety of the circumferential resection margin, surgeons should then operate in front of the DVF to ensure that the tumor can be resected radically with clear margins, even if there may be damage to the nerves or urogenital organs.

## Conclusion

According to those multifarious studies, we can give a general description of the DVF and its surrounding structures: posteriorly, the DVF is separated from the rectum by the prerectal space, which contains loose connective tissue; anteriorly, the DVF is adjacent to the prostate and seminal vesicle, and many communicating branches from the bilateral pelvic plexus run between them; bilaterally, the DVF may extend in the form of two layers, multiple layers, or fusion with the parietal pelvic fascia. No matter which architecture the DVF displays at the lateral side, it always keeps the neurovascular bundle away from the rectum. Although the controversies surrounding the DVF still confuse many surgeons and this general description may not be accurate, the authors hope that this review can at least provide some guidance for rectal surgery at present. In the future, further high-quality studies are required to help us to better understand the DVF.

## Authors’ contributions

X.M.Z. and G.Y.Y. contributes equally to this work and are considered co-first author. X.M.Z. and G.Y.Y. bring the idea for the article; X.M.Z., G.Y.Y., N.X.Z. and H.M.L. performed the literature search and analysis; X.M.Z., G.Y.Y., N.X.Z. and H.M.L. wrote the paper; H.F.G. and Z.L. made critical revisions related to important intellectual content of the manuscript; W.Z. supervised and approved the final version of the review.

## Funding

This work is supported by the foundation from 1. the Shenkang Hospital Developing Center of Shanghai, China. The Project of Frontier Technology in General Hospital (No. SHDC12016122); 2. 234 Climbing Discipline Program of first affiliated hospital of Naval Medical University (No. 2019YXK032).

## Conflicts of interest

None declared.
